# Data on the techno-economic and financial analyses of hybrid renewable energy systems in 634 Philippine off-grid islands

**DOI:** 10.1016/j.dib.2022.108485

**Published:** 2022-07-24

**Authors:** Michael T. Castro, Jethro Daniel A. Pascasio, Joey D. Ocon

**Affiliations:** a Laboratory of Electrochemical Engineering, Department of Chemical Engineering, University of the Philippines Diliman, Quezon City 1101, Philippines; b Energy Engineering Program, National Graduate School of Engineering, College of Engineering, University of the Philippines Diliman, Quezon City 1101, Philippines

**Keywords:** global horizontal irradiance, wind speed, load profile, levelized cost of electricity, net present value, internal rate of return, energy systems modeling

## Abstract

This data article contains the location, energy consumption, renewable energy potential, techno-economics, and profitability of hybrid renewable energy systems (HRES) in 634 Philippine off-grid islands. The HRES under consideration consists of solar photovoltaics, wind turbines, lithium-ion batteries, and diesel generators. The islands were identified from Google Maps™, Bing Maps™, and the study of Meschede and Ocon et al. (2019) [1]. The peak loads of these islands were acquired from National Power Corporation – Small Power Utilities Group (NPC-SPUG), if available, or estimated from the island population otherwise. Hourly-resolution load profiles were synthesized using the normalized profiles reported by Bertheau and Blechinger (2018) [2]. Existing diesel generators in the islands were compiled from reports by NPC-SPUG, while monthly average global horizontal irradiance and wind speeds were taken from the Phil-LIDAR 2 database. Islands that are electrically interconnected were lumped into one microgrid, so the 634 islands were grouped into 616 microgrids. The HRES were optimized using Island System LCOE_min_ Algorithm (ISLA), our in-house energy systems modeling tool, which sized the energy components to minimize the net present cost. The component sizes and corresponding techno-economic metrics of the optimized HRES in each microgrid are included in the dataset. In addition, the net present value, internal rate of return, payback period, and subsidy requirements of the microgrid are reported at five different electricity rates. This data is valuable for researchers, policymakers, and stakeholders who are working to provide sustainable energy access to off-grid communities. A comprehensive analysis of the data can be found in our article “Techno-economic and Financial Analyses of Hybrid Renewable Energy System Microgrids in 634 Philippine Off-grid Islands: Policy Implications on Public Subsidies and Private Investments” [3].

## Specifications Table

**Table:** 

Subject	Energy
Specific subject area	Renewable Energy, Sustainability, and the Environment
Type of data	Table
How the data were acquired	Government reports (from National Power Corporation – Small Power Utilities Group, Philippine Statistics Authority, and distribution utilities) Published journal articles Datasets (Facebook™ High Resolution Population Density, Phil-LIDAR 2) Software (ISLA, our in-house energy systems modeling tool).
Data format	Raw Analyzed
Description of data collection	Data on the 634 off-grid islands were compiled from government reports, published journal articles, and datasets. These were used as input into an energy systems modeling tool, which designs a least-cost hybrid renewable energy system in these islands. The techno-economic and profitability metrics of the optimized systems are also added to the dataset.
Data source location	Philippines
Data accessibility	Repository name: Mendeley Data Data identification number: 10.17632/cg5z25rj8y.1 Direct URL to data: http://dx.doi.org/10.17632/cg5z25rj8y.1
Related research article	M.T. Castro, J.D.A. Pascasio, L.L. Delina, P.H.M. Balite, J.D. Ocon, Techno-economic and financial analyses of hybrid renewable energy system microgrids in 634 Philippine off-grid islands : Policy implications on public subsidies and private investments, Energy. 257 (2022) 124599. https://doi.org/10.1016/j.energy.2022.124599.

## Value of the Data

• The data could be useful for policy making and energy planning because the techno-economic feasibility and profitability of hybrid renewable energy systems in off-grid islands is described in this dataset.
• The data benefits researchers who wish to build their own energy systems models as well as policymakers and stakeholders who need to make informed decisions about funding energy systems for off-grid communities.
• Studies that consider other renewable energy technologies, such as hydro and biomass, can build on the energy demand, global horizontal irradiance, and wind speed data in this article.
• Machine learning techniques can be applied on this dataset to investigate the transferability of off-grid energy systems between similar islands.

## 1 Data Description

Table S1 (see Microsoft Excel file with data) presents an initial list of 1050 Philippine islands, while Table S2 shows a filtered list of 634 residential Philippine off-grid islands. Table S3 details the electrical interconnections between the 634 off-grid islands and how these were grouped into 616 microgrids. The monthly peak consumption of some islands according to the National Power Corporation – Small Power Utilities Group (NPC-SPUG) from 2016 to 2019 is provided in Table S4, while the most recent energy consumption per month is summarized in Table S5. The peak energy demands of the off-grid islands are presented in Table S6. The normalized monthly and hourly profiles in Table S7 are used to generate hourly resolution load profiles from the peak load. Existing diesel generators in each microgrid are tabulated in Table S8, while the global horizontal irradiance (GHI) and wind speed in each microgrid are shown in Tables S9 and S10, respectively. Table S11 contains the techno-economic metrics of the cost-optimum hybrid renewable energy system (HRES) in each microgrid. The HRES consists of solar photovoltaics (PV), wind turbines, lithium-ion (Li-ion) batteries, existing diesel generators, and additional diesel generators. The techno-economic metrics reported in work are the optimum component sizes, net present cost (NPC), levelized cost of electricity (LCOE), total discounted capital, operating, replacement, and fuel costs, renewable energy share (RE-Share), annual fuel consumption, and CO_2_ emissions per kWh of electricity generated. Lastly, Table S12 summarizes the net present value (NPV), internal rate of return (IRR), payback period (PBP), and subsidy requirements of the cost-optimum HRES at five different electricity rates. First is the subsidized-approved generation rate (SAGR), which is what the electric cooperative (EC), which is in charge of power distribution, is required to pay NPC-SPUG for power generation. The SAGR is lower than the true cost of generation (TCGR) since the difference is subsidized through the universal charge for missionary electrification (UCME) scheme. Second is 0.2 USD/kWh, which is representative of the retail electricity rate paid by mainland residents. Third is a national breakeven rate at which the total NPV across all microgrids is zero. The last two electricity rates are the breakeven rate increased by 25% and 50%, respectively.

## 2 Experimental Design, Materials and Methods

### 2.1 Off-grid islands

The initial list of 1050 Philippine islands is composed of the 502 islands considered in the work of Meschede et al. (2019) [1] and 548 additional islands collected from Google Maps™ and Bing Maps™. Wholly non-residential island, stilts and shallow communities, and islands interconnected to the main grid are then excluded to form a list of 634 residential Philippine off-grid islands. Purely touristic islands were specified in the Department of Tourism's list of accredited establishments, while non-residential islands were manually identified in Google Maps™ based on the absence of dwelling structures. Stilts and shallow communities were also inferred from Google Maps™. NPC-SPUG has also documented which islands are interconnected to the main grid.

**Fig. 1:**
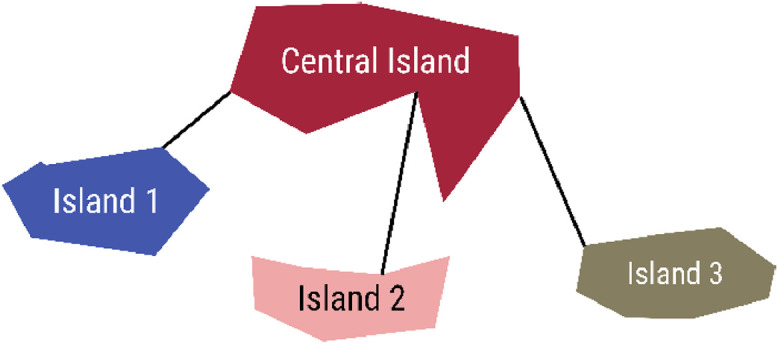
A cluster of interconnected islands lumped into one microgrid. The islands are all connected to one central island.

Some of the islands are electrically interconnected, so these were grouped into one microgrid. The interconnection structure of clustered islands in a microgrid is presented in Fig. 1. There is a central island that houses all existing generation units. The other islands in the cluster are interconnected to this central island. Islands that are not connected to any other island are also referred to as microgrids and are their own central island. The 634 off-grid islands are grouped into 616 microgrids.

### 2.2 Load profiles

The monthly peak power consumption of some islands has been recorded by NPC-SPUG from 2016 to 2019. There are some months when data is lacking, however. To create a representative monthly peak power consumption profile, the most recent peak consumption of each month is selected as the peak power in that month. For instance, if the consumption in January 2019 is unavailable, then the consumption in January 2018 is taken as the consumption in January. There are 99 islands with a complete set of monthly peak consumption data from NPC-SPUG and three additional islands wherein only the annual peak consumption is known.

**Fig. 2:**
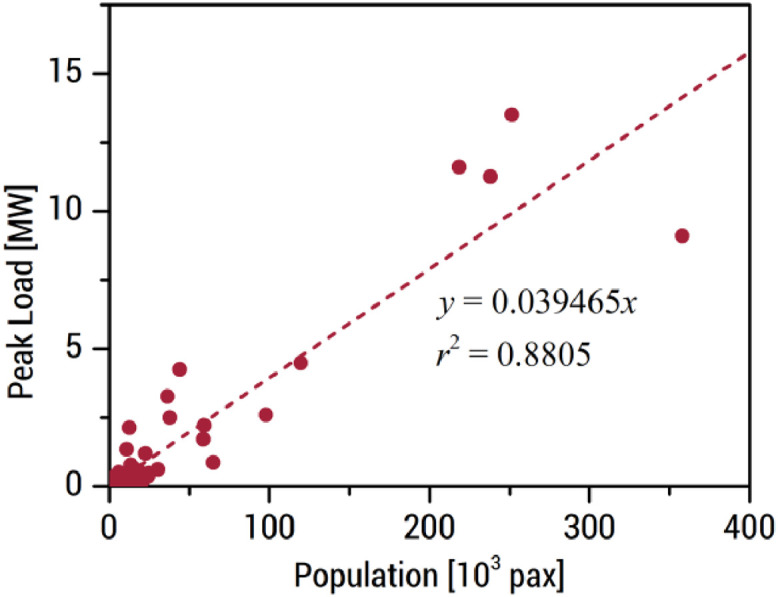
Annual peak load vs. population of the 102 islands with peak load data from NPC-SPUG. The proportional correlation developed here is used to estimate the peak load of the other islands without reported peak loads.

As for islands without actual consumption data, their annual peak loads are estimated based on the population. The population of the 102 islands with annual peak consumption data is determined either from the Philippine Statistics Authority (PSA), if available, or from Facebook™ High Resolution Population Density maps. A proportional correlation (i.e., best-fit line with zero intercept) is then developed between the peak load and population, as shown in Fig. 2. The population of the remaining 532 islands is estimated in the same manner as the 102 islands, and the peak load vs. population correlation is applied to determine the annual peak load.

The monthly and annual peak loads are converted into an hourly resolution using the normalized load profiles reported by Bertheau and Blechinger (2018) [2]. If the annual peak load is known, then it is multiplied with the normalized load profiles to generate hourly-resolution day-long load profiles for each month. If the monthly peak loads are known, then the hourly-resolution day-long load profiles of each month are scaled to match each of the monthly peak loads.

### 2.3 Existing diesel generators

The list of existing diesel generators in each microgrid was compiled from reports by NPC-SPUG [4], [5], [6]. Only operational diesel generators have been included in our dataset.

### 2.4 Renewable energy resources

The monthly average GHI and wind speeds were obtained from the Phil-LIDAR 2 database [7]. The original dataset primarily covered the mainland, so nearest neighbors interpolation was performed to obtain data at more distant islands. The resolutions of the original GHI and wind speed rasters were 10 m × 10 m and 5 km × 5 km, respectively, but the GHI data was resampled to 5 km × 5 km prior to interpolation to reduce computational costs. The GHI and wind speeds were evaluated at the centroid of the central island of each microgrid.

### 2.5 Techno-economic metrics

The techno-economic metrics describe the cost-optimum HRES consisting of solar PV, wind turbines, Li-ion batteries, existing diesel generators, and additional diesel generators in each microgrid. The HRES was modeled in Island Systems LCOE_min_ Algorithm (ISLA), our in-house energy systems modeling tool, which determines the sizes of the HRES components such that the NPC is minimized. The per-unit costs and lifespan of the HRES components, which were taken as input to ISLA, are presented in Table 1.

**Table 1:** Input techno-economic parameters to ISLA

Component	Parameter	Unit	Value	Ref.
Solar PV	Capital Expenses	USD/kW	1500	[8]
	Operating Expenses	USD/kW·y	15	[8]
	Lifetime	y	20	[8]

Wind	Capital Expenses	USD/kW	7900 (if < 31.6 kW) 5700 (if 31.6 – 316 kW) 1300 (if > 316 kW)	[†]
	Operating Expenses	USD/kW·y	1% of Capital Expenses	
	Lifetime	y	20	[8]

Li-ion	Capital Expenses [*]	USD/kW	700	[8]
	Operating Expenses	USD/kW·y	5	[8]
	Roundtrip Efficiency	%	90	[8]
	Lifetime	y	10	[8]

Diesel	Capital Expenses	USD/kW	500	[9]
	Variable Operating Expenses	USD/kWh	0.03	[10]
	Fuel Costs	USD/L	0.9	[11]
	CO_2_ Emissions	kg/L	2.67	[11]
	Lifetime	h	15000	[10]

Project	Capital Expenses	USD	0	
	Operating Expenses	USD/y	0	
	Discount Rate	%	8	[8]
	Lifetime	y	20	[8]

[*] For additional generators only. This is zero for existing generators.

[†] Based on a review of commercially available wind turbines.

### 2.6 Profitability metrics

The profitability metrics characterize the cost-optimum HRES under five different electricity rates. The NPV, IRR, and PBP are computed assuming that the annual profit is equal to the electricity rate multiplied by the annual energy consumption. The subsidy requirements are calculated based on the guidelines of the Energy Regulatory Commission [12,13]. These incentivize the generation of renewable energy and penalize excessive diesel use.

## CRediT Author Statement

**Michael T. Castro:** Conceptualization, Methodology, Software, Formal analysis, Data curation, Writing – original Draft, Visualization; **Jethro Daniel A. Pascasio:** Validation, Formal analysis, Data curation, Writing – review & editing; **Joey D. Ocon:** Conceptualization, Methodology, Resources, Writing – review & editing, Supervision, Funding acquisition.

## Declaration of Competing Interest

The authors declare that they have no known competing financial interests or personal relationships that could have appeared to influence the work reported in this paper.

The authors declare the following financial interests/personal relationships which may be considered as potential competing interests:

## Data Availability

Data on the techno-economic and financial analyses of hybrid renewable energy systems in 634 Philippine off-grid islands (Original data) (Mendeley Data).
